# Metastatic bone disease in proximal femur. Outcome of surgical treatments. − Do we know what to do?

**DOI:** 10.1016/j.jbo.2025.100711

**Published:** 2025-09-06

**Authors:** K. Kilk, G. Kask, J. Nieminen, M.K. Laitinen

**Affiliations:** a Department of Orthopaedics and Traumatology, Helsinki University Hospital and University of Helsinki, Helsinki, Finland; b Coxa, Hospital for Joint Replacement, Tampere, Finland

**Keywords:** Bone metastases, Metastatic fracture, Pathological fracture, Proximal femur, Impending fracture

## Abstract

**Background:**

Skeletal metastases related pathological fracture reconstruction methods in proximal femur range from osteosynthesis to tumor prostheses with acetabular reconstruction, depending on lesion size and location. This retrospective study, of 299 patients surgically treated for proximal femur metastases, investigates implant survival, complications, and functional outcomes of various surgical strategies for treating pathological fractures of the proximal femur.

**Patients and methods:**

This retrospective study of 299 patients surgically treated for proximal femur metastases, investigates implant survival (Kaplan–Meier), complications, and functional outcomes of different surgical strategies. The chi-test and Mann-Witney *U* test were used for analysis between groups. The subdistribution Hazard Ratio (SHR) of the role of factors affecting implant survival was calculated using competing risk analysis.

**Results:**

Reconstruction methods comprised osteosynthesis (n = 59), hemiarthroplasty (n = 72), total hip replacement (THA) (n = 43), and endoprosthetic replacement (EPR) either with or without acetabular component (n = 125). The precise location and size of the metastases was evaluated. The mean implant survival was 17 months (SD 21.2). Complications occurred in 33 patients, 20 required revision surgery. In prosthesis patients, infections and dislocations were the main complications, while mechanical failure predominated in the osteosynthesis group. Mean implant failure time was 11 months, shortest in THA and osteosynthesis. Functional outcomes in 38 patients showed a mean Oxford Hip Score (OHS) of 33, with no significant differences across methods.

**Interpretation:**

Patient survival is a critical factor in selecting the appropriate reconstruction method for trochanteric metastatic lesions. Osteosynthesis is suitable for patients with a limited life expectancy. In cases of metastases involving the head-neck anatomical region, arthroplasty with acetabular reconstruction offers no advantage over hemiarthroplasty. With our data there was no statistical difference in functional outcome between different surgical methods.

## Highlights

• Assessing patients’ life expectancy is key in choosing surgery method.
• Intramedullary nailing fits patients with short life expectancy.
• Acetabular reconstruction only when clinically indicated.
• No surgical method proved superior in functional outcomes.

## 1 Introduction

**Fig. 1:**
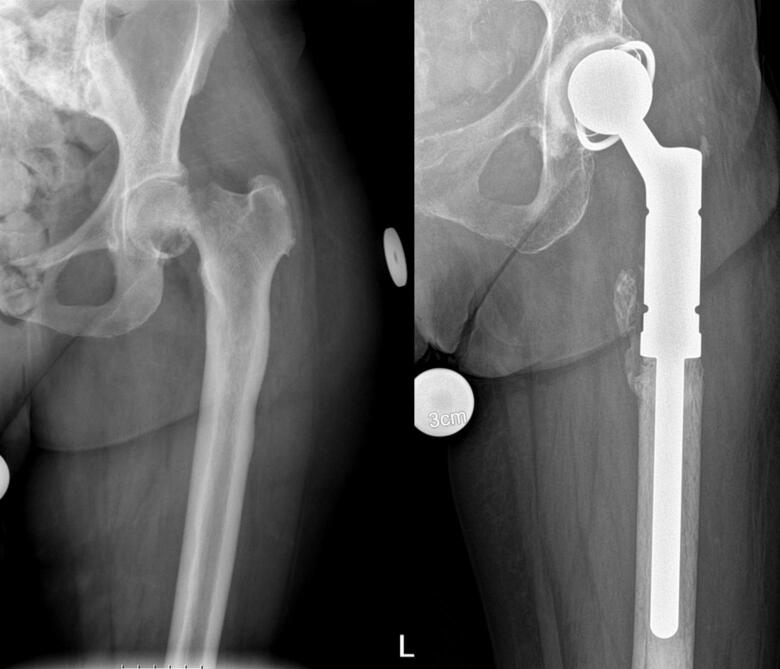
Pathological femoral neck fracture treated with endoprosthetic replacement (EPR).

Bone is the third most common site of metastatic disease, after the lung and liver [1]. In skeletal metastases, or metastatic bone disease (MBD), normal bone metabolism is disrupted, and healthy bone tissue is replaced by cancerous tissue, leading to weakened bone structure and an increased risk of pathological fractures [1,2]. Recent advances in systemic oncological therapies and supportive care have substantially extended survival for patients with MBD. A recent multi-institutional analysis reported that median survival after surgical treatment of MBD has significantly increased over the past two decades [3]. Similarly, population-level data from the United States highlight declining cancer mortality rates and increasing numbers of individuals living with advanced cancer, underscoring the growing cohort of patients surviving longer with skeletal metastases [4]. Although pathologic fractures frequently occur in the ribs and vertebrae, fractures of long bones and pelvis lead to severe disability, reduced quality of life, and often necessitate surgical procedures [1,2]. The proximal femur accounts for approximately 65 % of skeletal metastases requiring surgery [5,6]. A systemic literature review by Janssen et al highlighted a lack of scientific evidence regarding implant outcomes and revision risks for proximal femoral reconstructions [7]. Surgical treatment options for proximal femur metastatic lesions include osteosynthesis or prosthetic reconstruction, ranging from hemiarthroplasty to extensive resections with endoprosthetic (tumor prosthesis) replacement (EPR) (Fig. 1). To optimize quality of life (QoL), recovery from surgery should be shorter than expected survival, particularly in electively cases for impending pathologic fracture. The reconstruction should also permit full weight-bearing soon after the surgery ideally as a single revisions-free procedure [8]. Osteosynthesis enables rapid recovery and better short-term functional outcomes but carries a higher risk of mechanical failure in patients with longer survival. Cemented EPR enables early weight-bearing and shows good functional outcomes in primary bone tumor surgery [7,9,10]. Hemiarthroplasty, with unipolar or bipolar femoral head components, has a lower risk of dislocation compared to total hip arthroplasty (THA) [11]. While acetabular erosion from hemiarthroplasty increases over time, conversion to total acetabular reconstruction remains relatively uncommon. Lex et al., 2021; 103-B [12]:1633–40. The primary aim of this study was to evaluate implant survival and implant related complications, with a secondary aim to assess functional outcomes after reconstruction.

## 2 Patients and methods

We evaluated a total of 299 patients who underwent surgical treatment for either a complete or impending pathologic fracture in the proximal femur between 1/2000 and 12/2020 at Helsinki University Hospital and Tampere Coxa Hospital for Joint Replacement. Data was obtained from institutional surgical registries at Helsinki University Hospital and Coxa Hospital for Joint Replacement. Radiological assessment of relevant imaging was conducted to confirm eligibility. Patient and reconstruction-related outcomes were recorded. All patients over 18 years of age with histologically confirmed metastatic bone disease of any primary malignancy, including multiple myeloma and lymphoma, were included. Decisions regarding surgical intervention for fractures were discussed in a multidisciplinary tumor board. Prognostic considerations, including anticipated patient survival, were incorporated into the decision-making process, with clinical judgment informed by the principles of the SSG scoring system [5], although this was not formally applied as a standardized tool in the present cohort. Final treatment decisions were reached collaboratively between the treating physicians and the patient, integrating all relevant clinical factors.

**Fig. 2:**
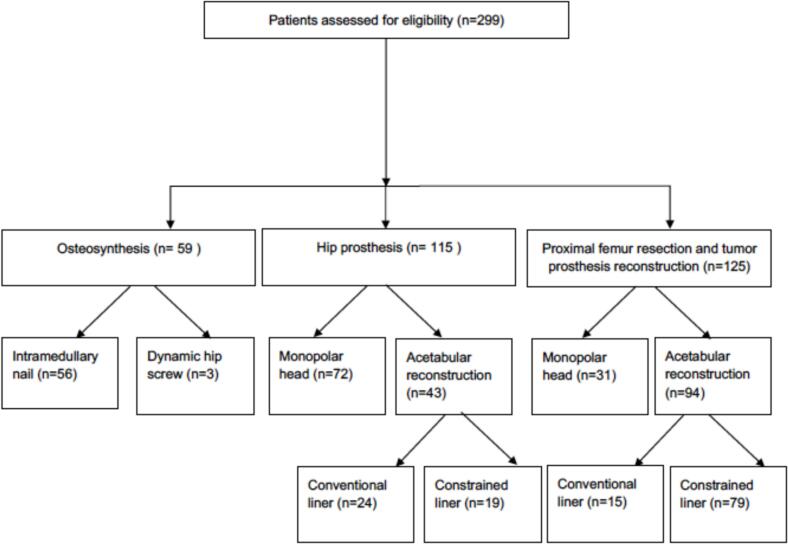
Operative methods.

Pre-operative radiological assessment included plain radiographs for all cases, with computed tomography (CT) or magnetic resonance imaging (MRI) performed in selected cases. Systemic staging included a CT scan of the chest, abdomen, and pelvis to assess the extent of metastatic disease. We evaluated the precise location and size of the metastases and categorized the proximal femur into three anatomical regions: A) head and neck B) trochanteric C) subtrochanteric area. Smaller metastases were confined to a single anatomical region, while larger metastases extended across multiple regions, with the largest metastases spanning from the femoral head to subtrochanteric area (Table 1). Surgical treatment methods included osteosynthesis with an intramedullary nail (IMN), hemiarthroplasty, THA, or EPR with or without acetabular reconstruction. In cases requiring acetabular reconstruction, both conventional and constrained liners were used. (Fig. 2). Postoperative complications, including mechanical complications, were classified according to the Henderson (2011) classification (Table 2) [13]. The classification system was also applied to the osteosynthesis group.

**Table 1:** Patient demographics.

Characteristics	Osteosynthesis	Hemi-arthroplasty	Total hip replacement	Endoprosthetic replacement	Total	p-value
Number of cases	59 (20 %)	72 (24 %)	43 (14 %)	125 (42 %)	299 (100 %)	
Sex						0.883
Male	23	33	18	52	126 (42 %)	
Female	36	39	25	73	173 (58 %)	
Mean age in years (±SD)	66 (14)	67 (13)	63 (13)	67 (11)	66 (12)	0.011
Side						0.423
Left	25	35	21	69	150 (50 %)	
Right	34	37	22	56	149 (50 %)	
True fracture	42	64	34	105	245 (82 %)	0.056
Impending	17	8	9	20	54 (18 %)	
Follow up (months)	22.6(SD 29.1)	13.0(SD 19.9)	23.9(SD 24.7)	19.3(SD 21.3)	19.1(SD 23.4)	0.027
Complications	12	7	2	12	33 (11 %)	0.061
Revision rate	9	2	1	8	20 (6.7 %)	0.018
Metastasis size cm (±SD)	7.6 (4.5)	5.6 (3.5)	5.1 (2.1)	8.6 (4.0)	7.2 (4.0)	0.129
Metastasis location						
A- Head and neck	3	33	59	4	99 (33 %)	<0.001
B- Trochanteric	30	30	7	42	80 (27 %)	
C- Subtrochanteric	16	16	0	35	52 (17 %)	
A + B	1	6	3	21	31 (10 %)	
B + C	7	0	2	12	21 (7 %)	
A + B + C	2	3	0	11	16 (5 %)99 %	
Primary malignancy	Breast		104	34.8 %		0.282
	Kidney		43	14.4 %		
	Myeloma		35	11.7 %		
	Prostate		29	9.7 %		
	Non-small cell lung ca		23	7.7 %		
	Lymphoma		13	4.3 %		
	Colon		12	4.0 %		
	Other		38	13.4 %		

±SD – standard deviation; (Tests: Crosstabs, Pearson Chi-Square test; Independent Samples Median Test was used in continues variables.

**Table 2:** Complications (Henderson 2011).

Characteristics	Osteosynthesis (n = 59)	Hemi-arthroplasty (n = 72)	Total hip replacement (n = 43)	Endoprosthetic replacement (n = 125)	Total (n = 299)	p-value	Revision
Complication	12 (20 %)	7 (9.7 %)	2 (4.7 %)	12 (9.6 %)	33 (11 %)	0.061	20 (6.7 %)
Soft tissue failure	0	1 (1.4 %)	0	1 (0.8 %)	2 (0.7 %)	<0.001	1 (0.3 %)
Structural/mechanical failure	11 (19 %)	4 (5.6 %)	0	5 (4 %)	20 (6.7 %)		12 (4.0 %)
Infection	1 (1.7 %)	2 (2.8 %)	0	6 (4.8 %)	9 (3.0 %)		6 (2.0 %)
Tumor progression	0	0	2 (4.7 %)	0	2 (0.7 %)		2 (1.6 %)

Functional outcomes were assessed using the Oxford Hip Score (OHS) [14], a widely used patient-reported outcome measure (PROM). The maximum achievable OHS score is 48, with scores over 41 considered excellent, 34–41 good, 27–33 fair, and under 27 indicating a poor outcome [15]. OHS questionnaires were administered to patients with a minimum follow-up of six months (169 patients). Of these, 38 patients attended follow-up visits and completed the questionnaire (Table 3). The remaining patients did not complete functional outcome assessment, mainly due to poor health or fatigue, absence of clinical need for follow-up at our center.

**Table 3:** OHS results are stratified by operative methods in patients with survival more than 6 months (38 of 169).

Characteristics	Number	Median	Range
Total	38/169	41	17–48
Osteosythesis	1/33	46	46–46
Hemiarthroplasty	2/29	35	27–43
THA	7/31	46	17–48
EPR	28/76	38.5	19–47

p-value 0.051 (KruskalWallis test).

OHS – Oxford Hip Score; THA – Total hip replacement; EPR – Endoprosthetic replacement.

Comorbidity status was routinely assessed preoperatively using the American Society of Anesthesiologists (ASA) score and was considered in clinical decision-making. Since all patients were classified as ASA 3–4, ASA status was not included in the statistical analysis.

### 2.1 Statistics

Patient and implant survival rates were assessed using the Kaplan-Meier methods with 95 % confidence intervals (CI). Between-group comparisons were performed using the log-rank test. Patient follow-up time was calculated from the date of surgery to the most recent follow-up date or the date of death. Implant survival was calculated from the date of surgery to revision surgery due to any cause. Continuous variables are reported as mean and standard deviation and between-group differences are analyzed using one-way ANOVA. The chi-squared test or Fisher’s exact test was used to compare variables between groups, and the Mann-Whitney *U* test for medians between two groups and Kruskal Wallis test between four groups. The subdistribution Hazard Ratio (SHR) of the role of factors affecting implant survival was calculated using competing risk analysis, where death was considered as a competing event. All statistical analyses were performed using SPSS Statistics 27.0 (IBM, New York, USA) but competing risk analyses were performed using STATA 16 (Stata, College Station, Texas, USA). No formal sample-size calculation was performed, as all eligible patients within the study period were included due to the retrospective design.

### 2.2 Ethics, funding, and potential conflicts of interest

This retrospective study was approved by the local chair of the audit department. This research study received a grant from the state funding for university-level health research. The funder had no role in study design, data collection and analysis, decision to publish, or preparation of the manuscript. No competing interests are declared.

## 3 Results

The study population comprised of 299 patients (173 women, 126 men) with a mean age of 66 years (SD ± 12.6 years) at the time of surgery. The mean follow-up period was 19 months (SD ± 23.4) and at the final follow-up, 43 patients (14 %) were alive. Overall patient survival was 57 % (169/299 patients) at 6 months and 43 % (128/299) at 1 year, with survival rates being similar across different surgical treatment groups. The most common primary malignancy was breast cancer (104/299, 35 %), followed by kidney (43/299, 14 %) and prostate cancer (29/299, 10 %). Surgery was indicated for impending fractures in 54 patients (18 %) while 245 patients (82 %) presented with complete pathological fractures. The mean size of metastatic lesion was 7.2 cm (SD ± 0.2; range, 2 to 30 cm) as measured by X-ray, CT, or MRI. (Table 1).

### 3.1 Implant survival and metastatic lesion evaluation

Overall patient and implant survival data are summarized in Fig. 3A, while survival stratified by operative method is presented in Fig. 3B. At 12-months, overall survival rates were as follows: osteosynthesis 44 % (95 %CI 31–57 %); hemiarthroplasty 27 % (95 %CI 17–33); THA 54 % (95 %CI 39–69); and EPR 52 % (95 %CI 43–61) (p = 0.027). At 12-months, implant survival rates were as follows: osteosynthesis 81 % (95 %CI 69–94 %); hemiarthroplasty 96 % (95 %CI 89–100); THA 97 % (95 %CI 91–100); and EPR 95 % (95 %CI 90–99) (p = 0.020).

**Fig. 3A:**
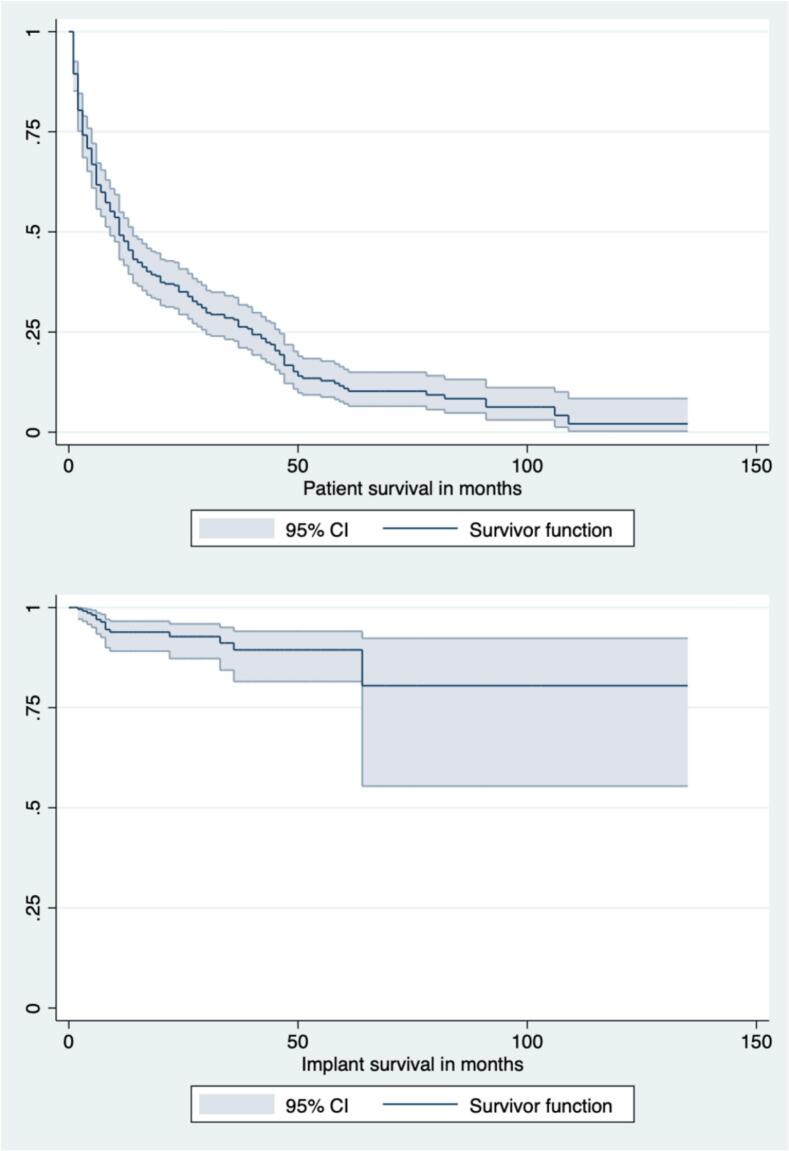
Overall patient and implant survival.

**Fig. 3B:**
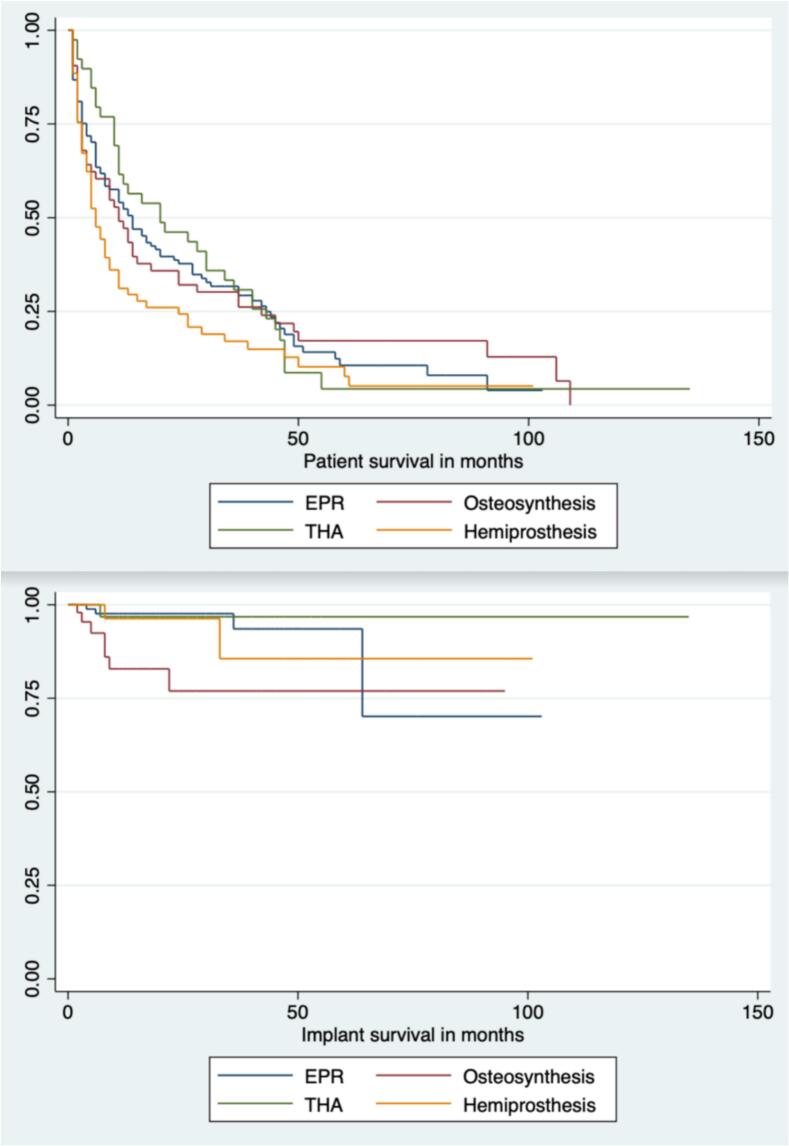
Patient and implant survival stratified by operative method. OHS – Oxford Hip Score; THA – Total hip replacement; EPR – Endoprosthetic replacement.

### 3.2 Complications

In total, 33 out of 299 patients (11 %) experienced postoperative complications, of which 20 cases (6.7 %) were treated with revision surgery. Deep infection occurred in 9 patients (9/33; 27 %), resulting in an overall infection rate of 3 % (9/299). (Table 2).

Conservatively treated complications included 6 prothesis dislocations managed with closed reduction, one postoperative hemorrhage managed by angiographic embolization and 6 cases that would have otherwise required a revision surgery but were managed conservatively due to poor overall condition.

Among arthroplasty patients, dislocation occurred in 7 patients (7/219; 3.2 %), with 6 cases managed by closed reduction. Dislocations were distributed as follows: 5 in the EPR group (4 total EPR − 2 with constrained liner and 2 with conventional liner and 1 hemi-EPR), 2 in the regular hemiarthroplasty group. No dislocations occurred in THA group. All constrained liners treated with closed reduction were of Freedom model (Zimmer Biomet, Warsaw, IN, USA). Acetabular protrusion requiring conversion to THA occurred only in 1 patient initially treated with hemiarthroplasty.

In the osteosynthesis group, complications requiring revision surgery were observed in 12 patients (12/59; 20 %). 11 out of 12 complications were osteosynthesis failure, with 1 failure occurring immediately and revised within days of the primary surgery. Revision surgeries included re-osteosynthesis in 3 cases and conversion to arthroplasty in 6 cases. Three complications in osteosynthesis group were managed conservatively due to patient’s poor overall condition.

### 3.3 Functional outcome

The first OHS measurements were performed after 6 months of follow-up. In this study, 169 of 299 patients (56 %) survived beyond 6 months and functional outcome measurements were available for 38 of 169 patients (23 %). The median overall follow-up time was 15 months (range, 6–116 months) (Table 3). The mean OHS score was 33 (range, 12–48), with the most functional outcome data collected from patients treated with arthroplasty (37/38), particularly EPR (28/38). Functional outcome data for the osteosynthesis group was limited, with Oxford Hip Scores (OHS) available only for one patient.

## 4 Discussion

### 4.1 Background

The surgical management of metastatic bone disease (MBD) in the proximal femur remains challenging due to the diversity of patient profiles, the varying anatomical extent of lesions, and the unpredictable prognosis of patients with skeletal metastases [7,16]. Although the proximal femur is the most frequent site requiring surgical intervention in cases of MBD, there is no established consensus on the optimal reconstruction technique.

Similarly to trauma surgery, the choice of reconstruction depends largely on the specific anatomical location of the lesion.

Pathologic fractures in the extracapsular region (trochanteric or subtrochanteric) are commonly treated with internal fixation, such as intramedullary nails (IMNs), especially in non-tumor centers. Nailing allows rapid recovery and shorter surgical times, which might be beneficial for patients with MBD and low residual life expectancy. Pathologic fractures in the head-neck area and large metastases necessitate some type of arthroplasty. Sturdier arthroplasty reconstruction is often chosen for MBD patients with higher life expectancy. Currently, there is no consensus on the optimal treatment for pathological metastatic fractures of the proximal femur. However, nearly all studies and guidelines emphasize that a patient's residual life expectancy is a critical factor to consider when selecting a reconstruction method. Predictive tools such as the Scandinavian Sarcoma Group (SSG) survival score exist, but accurately estimating life expectancy in patients with metastatic bone disease remains highly challenging [5]. This is reflected in the similar survival rates observed across reconstruction methods in our cohort, indicating that we have not been able to adequately predict residual life expectancy. This uncertainty hampers the ability to reliably select the most appropriate reconstruction method, as osteosynthesis is generally intended for patients with limited survival, while more extensive procedures such as tumor prostheses are typically reserved for those expected to live longer. The ongoing PERFORM prospective randomized trial is expected to provide valuable evidence by directly comparing fixation and reconstruction in metastatic femoral fractures and may ultimately offer clinicians more robust tools to match surgical strategy to patient survival [17,18].

### 4.2 Implant survival

In our study, the median patient age was 66 years, with a 1-year survival rate of 43 % and a follow-up period of 19 months. The distribution of primary malignancy was comparable to existing literature [6,[19], [20], [21], [22]].

The survival of osteosynthesis implants was the shortest among the evaluated methods; however, none of the reconstruction methods demonstrated improved survival based on the available data. Our observed revision rate of 6.7 % aligns with the rates reported in Jansen et al.’s systematic literature review (4.2 % for osteosynthesis with intramedullary nail and 5.2 % for prothesis) [21]. Notably, our revision rate of 6.7 % was slightly lower because some complications requiring surgical intervention were managed conservatively due to the patients' poor overall condition. If we include patients who required revision surgery but were treated conservatively, our true revision would have been 7.4 %.

Osteosynthesis for the treatment of pathological fractures in the proximal femur carries a higher risk of mechanical failure compared to more robust reconstruction with prosthesis. However, it allows for faster recovery and the opportunity to proceed with adjuvant treatment when necessary [23]. While our study focused on clinical outcomes, it is important to recognize that cost-effectiveness and resource utilization also play a major role in treatment selection. Differences in implant costs, operative time, and revision rates may influence overall health-system burden. These aspects warrant further prospective evaluation to better inform both clinical and policy-level decision-making. The risk of osteosynthesis mechanical failure is associated with the poor healing potential of pathological fractures and possible local tumor progression. Therefore, intramedullary nailing is considered a viable option for patients with a short life expectancy. In our study, 12 out of 59 patients treated with osteosynthesis experienced complications within a relatively short time frame (7.1 months). In 3 instances, patients were too compromised for revision surgery, suggesting that they benefited from the reduced surgical trauma and quicker recovery associated with osteosynthesis compared to prosthetic surgery. The remaining patients in the osteosynthesis group did not experience any complications.

### 4.3 Incidence and type of complications

In our analysis, the overall complication rate was 11 %, which aligns with findings from a similar study reporting a rate of 10.4 % [20].

It is hypothesized that patients undergoing hemiarthroplasty may experience a higher incidence of acetabular complications, such as hemiarthroplasty head protrusion into the acetabulum, and that hemiarthroplasties may be more prone to dislocation compared to prostheses with acetabular reconstruction featuring a locking ring.

In our cohort, which consisted solely of patients with proximal femur metastases, there was only one reported acetabular complication (acetabular protrusion) among the hemiarthroplasty patients (n = 103). This finding is supported by Meynard et al.'s study on surgical management for proximal femur metastasis, where no acetabular protrusion was reported in a group of 161 patients [20]. Preoperative planning and diagnostic assessments aimed at excluding hemiarthroplasty in patients with acetabular metastases likely contributed to the low rate of acetabular complications in our results. We recorded seven dislocations in our study, with no differences in dislocation rates based on the type of acetabular component (hemi/total or constrained/conventional). Majority of dislocations (six out of seven) were managed with closed reduction, while only one case required revision surgery. The Trevira attachment tube (Implantcast, Buxtehude, Germany) was utilized to enhance soft tissue reconstruction and joint stability in the tumor prosthesis group, irrespective of whether acetabular reconstruction was performed. Collectively, our results indicate that dislocation of prostheses is a rare complication, and neither the locking ring nor constrained liner completely prevents dislocation.

Lex et al. demonstrated in their study that there was no difference in acetabular complications between hemiarthroplasty and total EPR groups following EPR in patients with primary bone sarcomas and metastatic carcinoma. Lex et al., 2021; 103-B [12]:1633–40. Additionally, no differences in dislocation rates were found in a study comparing hemiarthroplasty and THA in patients with femoral neck fractures [24]. These findings support our conclusion that THA and total EPR are not superior to hemiarthroplasty and hemi-EPR concerning acetabular complications or postoperative instability. We conclude that hemiarthroplasty does not present a greater risk of acetabular complications, with acetabular protrusion being an uncommon occurrence. Furthermore, hemiarthroplasty helps avoid complications associated with acetabular reconstruction, such as perioperative issues and prolonged surgical time. There is no compelling reason to opt for acetabular reconstruction when it is unnecessary from the acetabular perspective.

### 4.4 Functional outcome

The first evaluation of functional outcome in our study occurred 6 months post-surgery. Notably, 130 patients (43 %) survived less than 6 months, highlighting the severity of MBD and its impact on the ability to gather comprehensive functional outcome data.

Data regarding functional outcomes in the osteosynthesis group are limited, as the Oxford Hip Score (OHS) was available for only one patient. Janssen et al. reported functional outcome scores following surgical treatment for proximal femur metastatic fractures, with mean MSTS scores ranging from 51 % to 74 % (based on 95 patients with follow-up from 6 to 27 months) and a TESS score of 67–71 % (follow-up from 18 to 27 months) after EPR, compared to 80 % after intramedullary nailing (24 patients, follow-up from 3 to 9 months) [21]. Similar to our findings, both our study and the review by Jansen et al. noted loss to follow-up in the osteosynthesis group, often due to patients being in poor overall condition or too fatigued to attend follow-up appointments [21].

Our functional outcome data yielded a median OHS score of 41 (range 17–48), indicating a generally good functional outcome [15]. Interestingly, we found no significant difference in functional outcomes across the various reconstruction methods, nor did we identify other factors such as age, BMI, or primary disease as relevant to functional outcomes. This lack of distinction may be attributed to additional factors affecting MBD patients, such as disease progression or side effects from chemotherapy and radiotherapy, which can influence assessments of functional outcomes and quality of life [25,26]. Potter et al. reported that patients with primary bone tumors exhibited statistically better functional outcomes than those with metastatic disease, with mean MSTS scores of 80.2 % and 66.8 %, respectively [10]. Consistent with our findings, preoperative pathological fractures did not appear to impact functional outcomes [10].

### 4.5 Limitations

This study is limited by its retrospective design, which introduces the possibility of selection bias despite the consecutive inclusion of patients. Furthermore, the incomplete PROM data reflects both the high mortality within the first six months, poor overall condition of patients with MBD and the absence of a systematic PROM collection protocol, which may have contributed to attrition bias. Additionally, in the prosthesis groups, functional outcomes were assessed using a protocol designed for arthrosis patients, resulting in functional outcome scores being available for only 37 patients treated with arthroplasty. These factors should be considered when interpreting the generalizability of our findings. However, our registry was meticulously maintained, and no patient data was lost to follow-up. Another limitation of this study is the application of the Henderson classification to osteosynthetic constructs, as it was originally designed for prosthetic reconstructions; however, given the lack of unified complication classification system covering both approaches, we chose Henderson for consistency, while acknowledging the need for a dedicated system for MBD patients.

## 5 Conclusions

This study underscores the importance of patient survival when determining the method of reconstruction for proximal femur trochanteric metastatic lesions. Intramedullary nailing appears to be a reasonable option for patients with limited life expectancy, conserving costs and resources while minimizing patient burden. In the femoral head and neck region, arthroplasty with acetabular reconstruction should be reserved for cases with a clear clinical indication. Within the limitations of this retrospective study, no surgical method showed clear superiority in terms of functional outcomes.

## CRediT authorship contribution statement

**K. Kilk:** Writing – original draft, Investigation, Data curation. **G. Kask:** Writing – review & editing, Supervision, Methodology, Data curation. **J. Nieminen:** Writing – review & editing, Supervision. **M.K. Laitinen:** Writing – review & editing, Supervision, Methodology, Conceptualization.

## Funding

This study was supported by state funding for university-level health research. The funder had no role in study design, data collection, analysis, or manuscript preparation.

## Declaration of competing interest

The authors declare that they have no known competing financial interests or personal relationships that could have appeared to influence the work reported in this paper.
